# P59 Multicentric osteolysis nodulosis and arthropathy syndrome: a case report in Erbil - Iraq

**DOI:** 10.1093/rap/rkae117.090

**Published:** 2024-11-05

**Authors:** Niaz Albarzinji, Aryan Jalal

**Affiliations:** College of Medicine, Erbil, Iraq; Kurdistan Higher Council for Medical Specialties, Erbil, Iraq

## Abstract

**Introduction:**

Multicentric osteolysis nodulosis and arthropathy (MONA) is inherited in an autosomal recessive manner. At conception, each sib of an affected individual has a 25% chance of being affected, a 50% chance of being an asymptomatic carrier, and a 25% chance of being unaffected and not a carrier. Once the MMP2 pathogenic variants have been identified in an affected family member, it is a skeletal dysplasia characterized by progressive osteolysis (particularly of the carpal and tarsal bones), osteoporosis, subcutaneous nodules on the palms and soles, and progressive arthropathy (joint contractures, pain, swelling, and stiffness).

**Case description:**

The patient is a 15-year-old female living in Erbil, Kurdistan. Her condition started when she was 10 months old, as she began walking and experienced recurrent sore throat. At age 4 year, she had a history of falling, developed stiffness and contracture in her elbow that made it difficult to move. A few months later, she developed a painful swelling/mass in her left foot that interfered with her walking. She had delayed tooth eruption, with only 2 teeth appearing per 2 years, her fontanelle did not close until age 3 years. At age 4, she had a fall that caused stiffness in her right hand. Over the next few years, she developed masses/swelling and pain/stiffness in her feet, hands, elbows, knees, and ankles, leading to deformities. She did not have any hearing problems but missed 3 years of school due to her condition. Back pain started 3 years ago and has been decreasing

Family History: Her sister, at age 4, had pain and mild swelling in her hands and feet, along with facial abnormalities. Another 4-year-old child in the family was admitted to the ICU with similar symptoms.

Complete blood count, serum biochemistry, rheumatoid profile was normal. Ultrasound of abdomen and ECHO was normal. On radiological imaging done for hands, feet, spine, in which in hands has diffuse generalized osteopenia, decrease joint spaces, resorptions of the phalanges, or cortical thinning and expansion of the phalangeal, metacarpal and metatarsal bone with deformity, and in x-ray of spine shows osteopenia with scoliosis. Dexa scan done for hip and spine which reveals (-6) severe osteoporosis.

The results of genetic testing on 26th of October, led to the diagnosis of MONA syndrome. The patient was started on supportive treatment (alendronate tablet 70 mg per week, Vit D tablet 5000 IU per day, NSAID for pain and physiotherapy).

**Discussion:**

The existing literature on MONA syndrome has broadly characterized the skeletal manifestations in the hands and feet. However, more detailed information is needed regarding specific skeletal involvement, ray involvement (which fingers/toes are affected), type and number of joints affected, laterality (whether the involvement is symmetrical or asymmetrical), type and severity of deformities, characteristic sparing of individual joints, if any longitudinal studies could help track the chronological order of joint involvement, especially in the larger or proximal joint

Research on MONA syndrome has described the skeletal manifestations in the hands and feet, but further study is needed to understand the specifics of ray involvement, joint involvement, laterality, and severity of deformity. Longitudinal studies can help track the progression of joint involvement, particularly in larger joints. The recurrence of symptoms after orthopedic surgery in one patient highlights the progressive nature of the syndrome and suggests that early surgical intervention may not be effective. Due to the rarity of MONA syndrome, it is difficult to fully understand the pattern of the syndrome’s phenotype. Furthermore, reported correlations between the syndrome’s genetic and phenotypic characteristics have been inconsistent and inconclusive

The treatment for MONA syndrome is primarily supportive and includes physical therapy to slow the development of contractures and prolong mobility. Pain relief may not be achieved through medication unless the pain is caused by secondary osteoarthritis or contractures. Additionally, daily recommended supplementation of vitamin D and calcium; standard management of congenital heart defects; management of scoliosis and kyphosis per orthopedist.

**Key learning points:**

• The complicated diagnostic process for MONA syndrome necessitates a re-review of the reported phenotype-genotype correlations. By expanding the understanding of the phenotype, including the detailed skeletal manifestations, the goal is to reach a consensus on the diagnostic criteria for MONA syndrome. The aim of this case report was to systematically describe the clinical manifestations, with a focus on the skeletal features, in children with molecularly confirmed MONA syndrome, and to establish relevant phenotype-genotype correlations.

